# Study on coupling effect of soil structure and overconsolidation on mechanical properties of loess

**DOI:** 10.1371/journal.pone.0298653

**Published:** 2024-03-13

**Authors:** Dequan Kong, Hailong Li, Songda Zhang, Feijie Qu, Rong Wan, Zhengchao Han, Shuai Li, Muhammad Usama Ahmad Khan

**Affiliations:** School of Civil Engineering, Chang’an University, Xi’an, Shaanxi province, China; University of Sharjah, UNITED ARAB EMIRATES

## Abstract

Soil structure and overconsolidation are two important factors that affect soil strength. Current research studies have primarily focused on the influence of single factors, and relatively few studies have studied the coupling effect of the two. In this paper, the effects of structure and overconsolidation on the mechanical properties of loess under certain conditions have been studied by compression tests and direct shear tests. Undisturbed loess, remolded loess, overconsolidated undisturbed loess, and overconsolidated remolded loess were investigated in this work. The results indicate that structure and overconsolidation can enhance the overall strength of the soil, but the effects of these two factors also interfere and weaken each other. The combined effect of structure and overconsolidation can lead to higher soil shear strength. Compared with remolded normally consolidated soil, when the vertical pressure is 50kPa, 100kPa, and 200kPa, the structure increases the strength of the original normally consolidated soil by 35%, 21%, and 7%, respectively. Overconsolidation increases the strength of the remolded overconsolidated soil by 51.3%, 40.9%, and 17.7%, respectively. The combined effect of structure and overconsolidation increases the strength of the original overconsolidated soil by 89%, 72.5%, and 32.7%, respectively. The increase in soil strength caused by the coupling effect is smaller than the sum of the strength increase caused by the two factors. The main reason is that the soil structure can reduces the compaction effect of overconsolidation, and the compaction load applied during the process of overconsolidation can also damage the soil structure, and the scanning electron microscopy observation is consistent with the experimental results and analysis. Finally, an empirical relation was developed for the effect of overconsolidation, structural properties, and their coupling on soil strength. The calculated results of the formula are highly consistent with the experimental data, and have good rationality and accuracy.

## 1 Introduction

Loess is a soil type with special engineering properties which is widely distributed worldwide. The experimental and theoretical research of loess has always been a focus and hotspot in the geotechnical field. The loess in China is mainly distributed in the western and northern regions, and in recent years, a large number of important basic engineering projects have been carried out in these areas, which has also brought many new opportunities and challenges to loess research. It has been shown that the strength of loess consists of two independent parts, consolidation strength and structural strength [1]. Many scholars have studied the structural and overconsolidation properties of soil respectively, however, there are limited studies on the coupling effects of these two properties on the mechanical characteristics of loess.

Loess is a sediment in the arid and semi-arid western region. Due to its specific generation and historical environments, it has its own distinct and unique structure, which has become one of the most fundamental internal factors affecting the mechanical properties of loess. There is a consensus on the importance of soil structure to its engineering properties. It is more appropriate to further study the soil structure to better understand the core issues of soil mechanics in the twenty-first century [2]. The importance of research on soil structure was first proposed by Terzaghi in 1943, and his study of soil structure was also the beginning of the study of soil microstructure [3]. Since then, a great deal of research has been carried out by scholars on the structural properties of soils from a microscopic point of view. Munoz Castelblanco et al. and Garcia Giménez et al. conducted scanning electron microscopy test on loess in France, Spain, and other regions, respectively, to analyze the microstructure characteristics of loess. The results has shown that loess is composed of grains and corresponding intergranular pore groups, and the microstructure of small pore areas in loess is sensitive to changes in water content and is prone to collapse and collapse behavior [4–6]. In addition, the creep deformation of loess is closely related to its microstructure, and the maximum coverage area of mesopores is the most important factor leading to creep deformation [7]. Nguyen et al. found that volumetric moisture content has a significant impact on soil thermal conductivity, the thermal conductivity of loess depends on the amount of water but not on its distribution within the soil microstructure in the suction range studied [8]. The deterioration process of undisturbed loess was inevitably accompanied by the generation and development of microscopic cracks within the specimen [9]. In general, intergranular carbonate and clay aggregates give loess its high strength in natural state [10]. However, when the water content of loess increases, the disintegration of clay agglomerates (cement), the disintegration of carbonate cement, and the disintegration of other cement triggers the disruption of the soil structure, and then, with the movement and rearrangement of the particles, the initial open structure is transformed into a closer one, and the strength of the soil body decreases rapidly, which eventually leads to an abrupt, discontinuous, and irreversible collapsing deformation [11–14]. In addition, soil structure is widely recognized to play a role in controlling the mechanical behavior of loess [15–17], it has been shown that the peak shear strength and strength parameters of loess are significantly reduced after the structural damage of loess [18], and that undisturbed loess had a greater shear stiffness under initial axial strain [19, 20]. The main reason is that under the action of shear force, fine particles easily enter the pores of large particles, and all the bonding force, sliding friction force, and biting force between coarse and fine particles are smaller than those between coarse particles. The microstructure near the interface between coarse and fine particles is also easily destroyed [21]. Some scholars have also studied the mechanical differences between original loess and remodeled loess [22, 23], the strength of original loess is greater than that of remodeled loess under the same conditions. As the confining pressure increases, the stress-strain curves of the two converge, and the effect of structural characteristics on the strength of loess is weakened. Georgiannou et al showed that the response of remodeled soil can be used as a reference for identifying the effect of structural properties on the compressibility and strength of undisturbed soil [24]. It can be seen that the mechanical properties of soil are closely related to its structural changes, and it is imperative to study the structural properties of loess.

Soil is a product of various geological processes with complex mechanical properties and usually exhibits varying degrees of consolidation. At present, the urbanization of loess areas in China is rapidly developing. Deep excavations, demolishing old structures, and building new structures are very common in such areas [25]. In some cases, the loess also exhibits an overconsolidated state, significantly affecting its strength and deformation. Original loess usually has more connected pores than compacted loess. Even if the dry density is the same, compacted loess still has a different pore structure from the original loess [26]. The consolidation state of soil has a great influence on its strength and deformation, understanding the mechanical behavior of compacted loess is of great significance for engineering construction in loess areas. Some researchers have been studied the deformation characteristics [27–30] and mechanical properties of unsaturated overconsolidated soils [31–33]. The process of overconsolidation in loess produces a large number of agglomerates, which may enhance the interlocking in the compacted loess and hence result in a relatively large friction angle, with the compaction, the pores in the loess sample change from macropores into mesopores and then to small pores, the pore space of the orientation of the distribution and the morphology of the complexity of the weakening of the soil to resist the deformation of the ability to enhance. The structural properties of the soil need to be explicitly taken into account during the loading phase [34]. Soil shear strength monotonically increases with the overconsolidation ratio [35, 36], the stress-strain relationship of normally consolidated and overconsolidated loess has strain-softening characteristics, and both peak and residual strengths increase with increasing perimeter pressure [37, 38]. Wang et al. found that the reconsolidation degree significantly affects the trend of the excess pore water pressure ratio changing with the increase in the cycle number of loads. The reconsolidation process after liquefaction of tailings will improve its liquefaction resistance [39]. In addition, some scholars have considered the structural and overconsolidation characteristics of soil in their constitutive models. Liu and Carter introduced three new parameters to describe the structure of clay, thus constructing a modified Cambridge model that considers the structure [40]. Mu et al. proposed a new elastic-plastic model for isotropic structured soil under saturated and unsaturated conditions, which can effectively simulate the initial structure of soil and its evolution under water load [41]. At present, the constitutive models related to overconsolidation and structure still need to be improved and developed, and more experimental research is needed to provide a basis and reference for establishing constitutive models.

The literature review shows that the influence of structural and overconsolidation characteristics on the mechanical properties of soil is still a hot topic at present, especially for loess, a special soil with significant structural characteristics. Considering the complex mechanism of over consolidation and structural interaction on loess, the correlation between the two is still unclear. However, engineering projects often encounter structural soil with a certain degree of overconsolidation, for example, unloading and reloading in projects such as demolishing old buildings to build new ones or deep excavations. Therefore, it is imperative to research the coupling effect of structure and overconsolidation on the mechanical properties of loess, which can guide practical engineering. It can also provide an experimental reference for the loess constitutive model considering the influence of overconsolidation and structure. Thus, the current study is an interesting subject with practical implications.

## 2 Materials and experimental methods

The structure and overconsolidation of soil can significantly influences its mechanical properties. In this paper, the compression test and rebound test were carried out on the undisturbed and remolded loess samples. The undisturbed sample was taken from the site and retained a relatively intact structure, while the remolded sample almost lost its structure due to significant disturbance. The corresponding deformation characteristics were analyzed and compared to study the influence of overconsolidation and structure on the compression deformation characteristics of loess. Direct shear tests were also carried out on undisturbed loess, remolded loess, overconsolidated undisturbed loess, and overconsolidated remolded loess. The corresponding strength characteristics were compared and analyzed. The coupling effects of structure and overconsolidation on the stress-strain relationship of loess were studied. And the mechanism was discussed in combination with electron microscopy experiments.

### 2.1 Test materials

The soil sample used in this experiment was taken from a construction site in the southern suburbs of Xi’an. The depth from which the soil was taken was about 2.5m – 3.5m. The soil sample was brownish-yellow with large and needle-like pores visible to the naked eye. The soil sample was in a hard-plastic state, had higher compressibility, and belonged to Q_3_ loess. The basic physical properties of soil samples are given in Table 1.

**Table 1:** Physical properties of loess samples.

Soil sample	Specific gravity	Moisture content/%	Natural density g/cm^3^	Void ratio	Liquid limit /%	Plastic limit/%
Q_3_ loess	2.71	21.5	1.557	1.115	31.4	19.1

The initial consolidation pressure, *p*_*c*_, of undisturbed loess can be determined by restoring the confining compression curve of the remolded soil and then using the Casagrande method on the restored compression curve. The preconsolidation pressure value of the soil sample used in this experiment is *p*_*c*_ = 120.18*kPa*.

### 2.2 Sample preparation and testing equipment

#### 2.2.1 Soil sample preparation

1. Preparation of undisturbed soil samples
  A ring knife of 6.18 cm diameter and 2.0 cm height was selected to prepare the original soil sample. The prepared soil sample was wrapped in plastic film and stored in a curing tank. The density difference of the same group of samples was controlled within 0.03 g/cm^3^, and the moisture content difference was less than 2% [42].
2. Preparation of remolded soil samples
  The original loess sample was first dried. It was then crushed and stirred with water, which was subsequently sealed and wrapped in a plastic bag. The sample was then put into a sample press for pressing. The density difference of the same group of samples was less than 0.02 g/cm^3^, and the difference in moisture content was less than 1% [42].

The undisturbed and remolded overconsolidated soil samples were tested by conducting lateral compression and rebound tests on the undisturbed and remolded soil samples, respectively, as given in Table 2. The nomenclature yz, yc, cz, and cc represent the undisturbed normally consolidated loess sample, the undisturbed overconsolidated loess sample, the remolded normally consolidated loess sample, and remolded overconsolidated loess sample, respectively. The remolded normally consolidated loess sample and the undisturbed normally consolidated loess sample are simply referred to as the remolded loess sample and the undisturbed loess sample.

**Table 2:** Details of samples and test methods.

Test methods	Sample types	Vertical pressure / kPa
Lateral compression Rebound test	undisturbed soil	1→12.5→25→50→100→200→400→800→1600→3200→1600→400→100→25→1
	remolded soil	
Lateral compression–Rebound—compression test	undisturbed soil	1→12.5→25→50→100→200→400→200→100→50→25→50→100→200→400→800→1600→3200→1600→400→100→25→1
	remolded soil	
Direct shear test	yz	50 100 200 400
	cz	50 100 200 400
	yc	50 100 200 600
	cc	50 100 200 600

#### 2.2.2 Test equipment

The unsaturated soil strain-controlled high-pressure consolidation straight shear (model: 4FDJ-20) from Jiangsu Liyang Yongchang Engineering Experimental Instrument Factory was used for testing. The main technical characteristics of the testing equipment are given in Table 3.

**Table 3:** Main technical indicators of the instrument.

Sample size /(cm^2^)	Normal pressure / kPa	Horizontal shear / kN	Counterpressure / kPa	Shear speed / (mm/min)
*φ*6.18×2	*P* = 0–4000	*F* = 0–6	*P*_*b*_ = 0–1000	V = 0.0024–1.2

### 2.3 Test arrangement

The specific experimental arrangement is given as follows.

#### 2.3.1 Confined compression test and rebound test

The confined compression-rebound test was performed on undisturbed and remolded loess samples with the same moisture content and initial pore ratio. The corresponding compression- rebound curve of the sample was obtained.

The compression-rebound-compression test was carried out on the undisturbed and remolded samples. The samples were first compressed to 400kPa and then rebounded to 25kPa. These were then compressed to 3200kPa and again allowed to rebound. The pressure of all levels of consolidation rebound was equal to that of confined compression, and the compression-rebound-compression curve was obtained.

These tests mainly compare the deformation characteristics of undisturbed and remolded loess. Also, the deformation characteristics of overconsolidated undisturbed loess and overconsolidated remolded loess can be compared.

#### 2.3.2 Scanning electron microscope test

The study also utilized scanning electron microscopy (SEM) to further explore the microstructure of undisturbed loess samples, compressed rebound soil samples, and compressed rebound recompressed soil samples from a microscopic perspective.

Before the experiment, soil samples were taken from fresh cross-sections and were air dried, The loose soil debris on the surface of the samples were blown away, and gold was sprayed on the soil particles with conductive glue. Following the above-mentioned steps, the gold sprayed samples were placed into a scanning electron microscope for observation (500x, 1000x, and 2000x).

#### 2.3.3 Direct shear test

Direct shear tests were conducted on yz, cz, yc, and cc samples. The consolidation slow shear method was used, and the shear rate was 0.0032mm/min. If there was a peak in the shear curve, the shear strength was taken as the peak, and if there was no peak, the shear stress corresponding to the shear displacement of 4mm was taken as the shear strength. The testing was carried out in two ways.

1. The original loess sample was loaded into the quadruple container chamber of the instrument, and the consolidation pressures were selected as 50kPa, 100kPa, 200kPa, and 400kPa, respectively. The direct shear test was carried out after consolidation. The above test was repeated for the remolded loess sample as well.
2. The original loess samples were loaded into the quadruple container chamber of the instrument. The consolidation pressure *p*_*x*0_ = 600*kPa* was applied to the samples first, and *p*_*x*0_ was used to represent the pre-consolidation pressure of the overconsolidated soil. The consolidation pressure was unchanged for one sample kept, whereas for the other three samples, it was 200kPa, 100kPa, and 50kPa, respectively, to artificially produce overconsolidation samples. A direct shear test was carried out after the rebound stability of the samples. The above tests were repeated with remolded loess samples. Correspondingly, the influence of structural and overconsolidation factors on the stress-strain relationship of loess was compared and analyzed for yz, cz, yc, and cc samples.

## 3 Results and discussion

### 3.1 Confined compression test and rebound test

**Fig 1:**
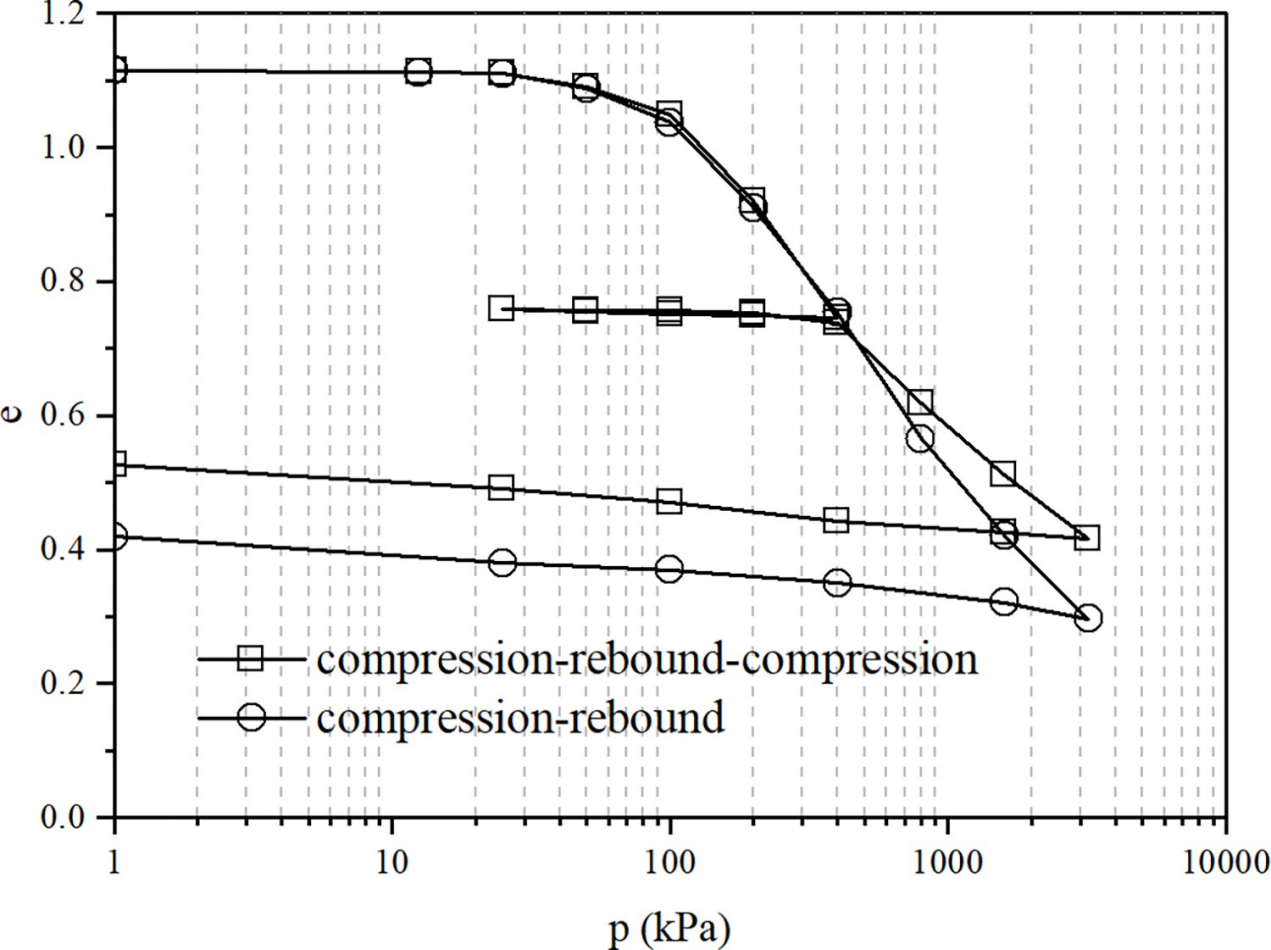
The compression-rebound and compression-rebound-compression test curves of undisturbed soil.

**Fig 2:**
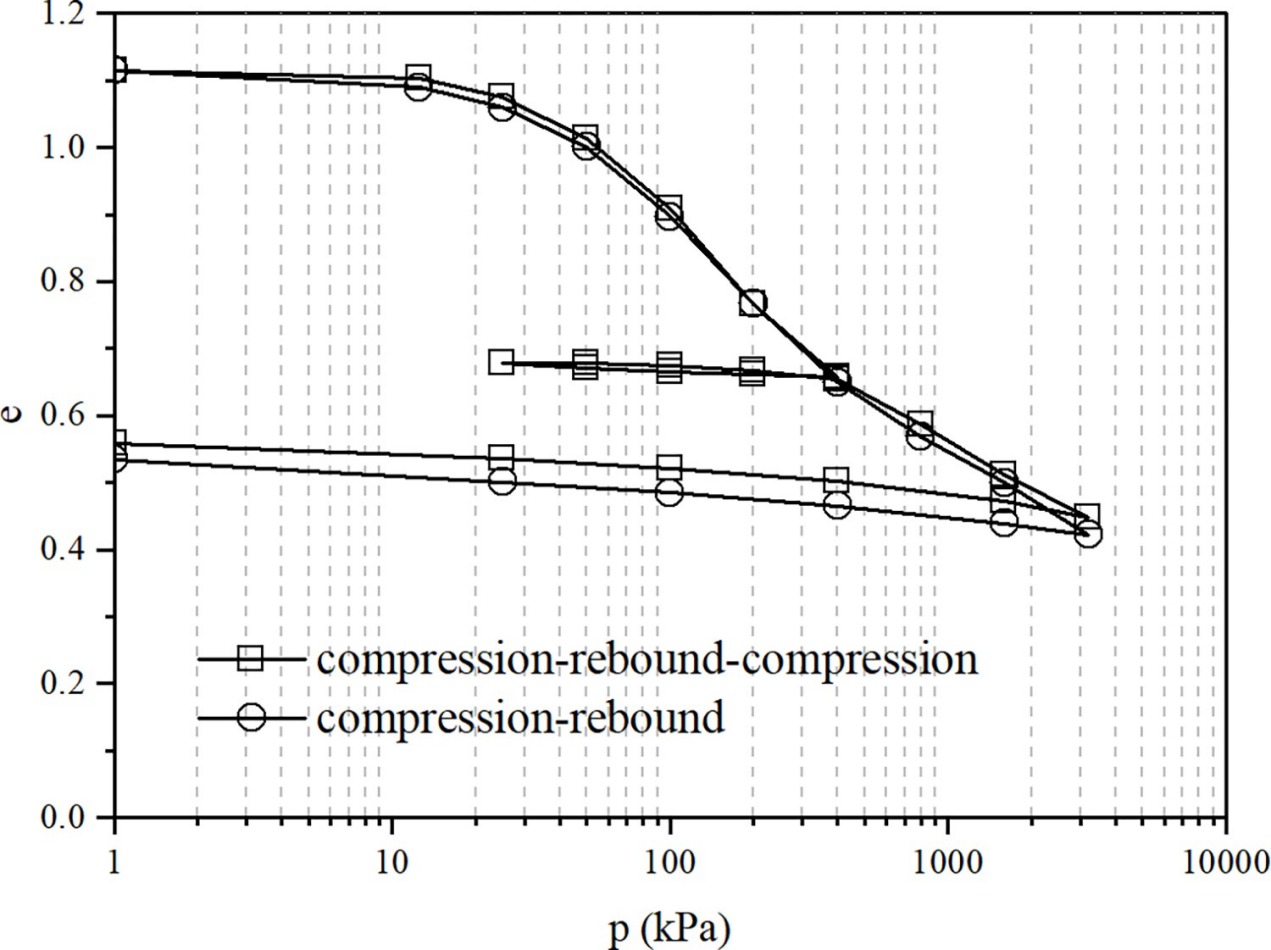
The compression-rebound and compression-rebound-compression test curves of remolded soil.

The compression-rebound and compression-rebound-compression test curves of the undisturbed soil sample are shown in Fig 1, while the compression-rebound and compression-rebound-compression test curves of the remolded soil sample are shown in Fig 2.

Figs 1 and 2 show that the compression-rebound curve and the compression-rebound- compression curve of undisturbed soil overlap when the vertical pressure is less than 400kPa, and the compressive deformation performance of the soil is the same. When the pressure exceeds 400kPa, the compression-rebound- compression sample has less deformation than the compression-rebound sample, and the compression coefficient and compression index also become smaller accordingly. The compressive performance of the soil has been improved to some extent during the rebound-recompression process. The test pattern for the remolded samples is similar to that of the undisturbed samples. Nevertheless, the recovery of the compressive properties of the remolded samples is less than that of the undisturbed samples (only 30.5% of the undisturbed soil), mainly because the undisturbed samples are more structural and have less deformation during loading. The soil structure recovery during unloading and rebound is greater than that of the remolded samples.

The connection and arrangement of the structure of loess affect the resulting properties. When the soil sample is unloaded and rebounded, the failure of some structural connections is irreversible, such as the failure of adhesive connections between soil particles. However, some structural connections have a certain degree of reversibility and can be restored after damage, such as the connection force generated by the double electric layer around loess particles and the suction force at the water-air interface. Loess is a material with large aggregates and pores in terms of arrangement and structure. Some of the aggregates are crushed during compression and fall into the pores. The rebound process adjusts the relative position of the particles, changes the size distribution of the pores, and leaves the soil sample in a more stable and denser state, which is conducive to enhanced soil strength and reduced deformation.

The undisturbed sample is in a state of overconsolidation after compression rebound, the structure is also restored, and the compressive performance is enhanced. The unloading and rebound process of soil is necessary for structural recovery and adjustment, and continuous loading causes the soil structure to be continuously damaged. Hence, the deformation of the compression-rebound sample is large, and the compressive performance is poor.

**Fig 3:**
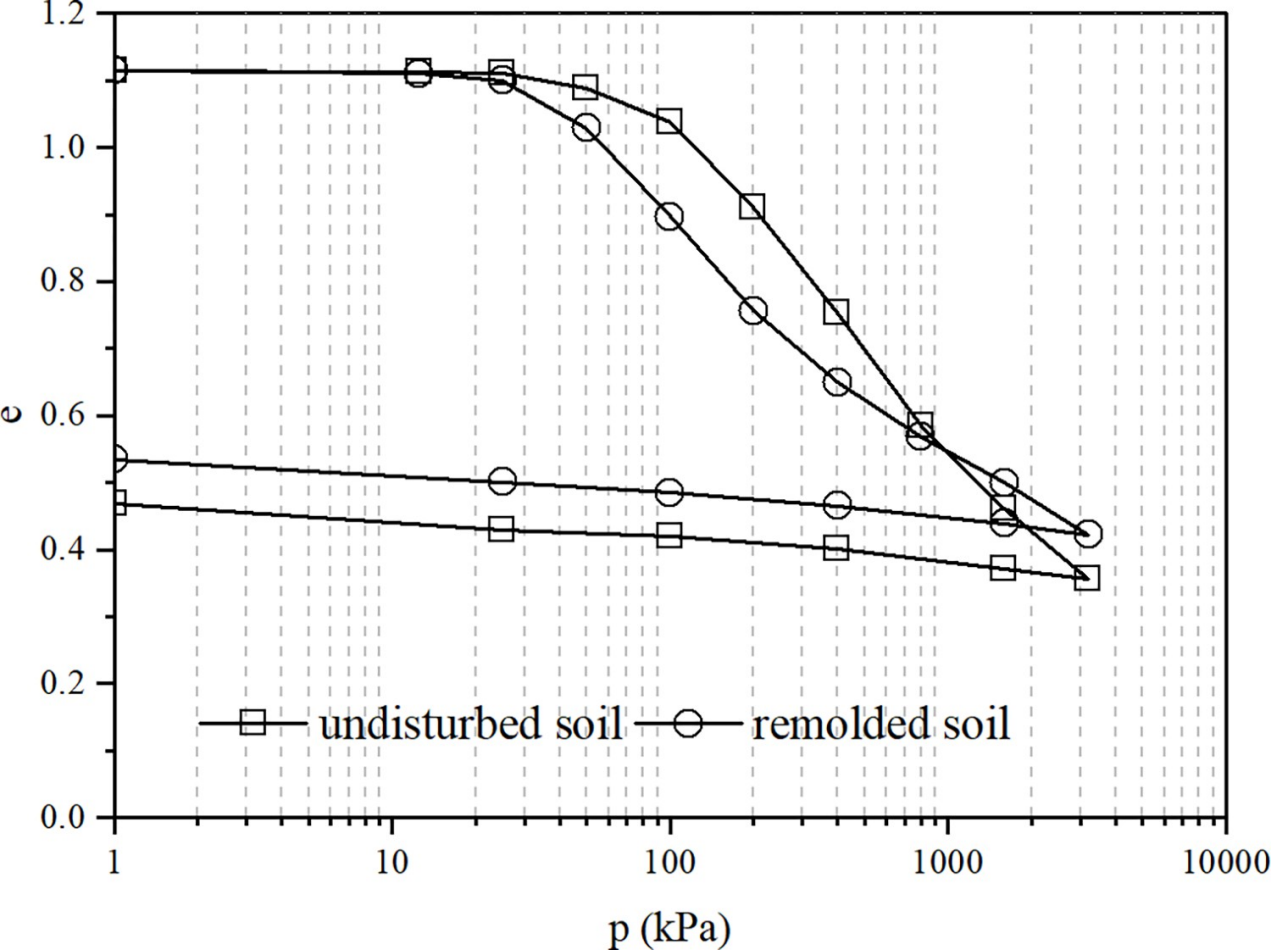
The compression-rebound test curves of undisturbed and remolded soil.

**Fig 4:**
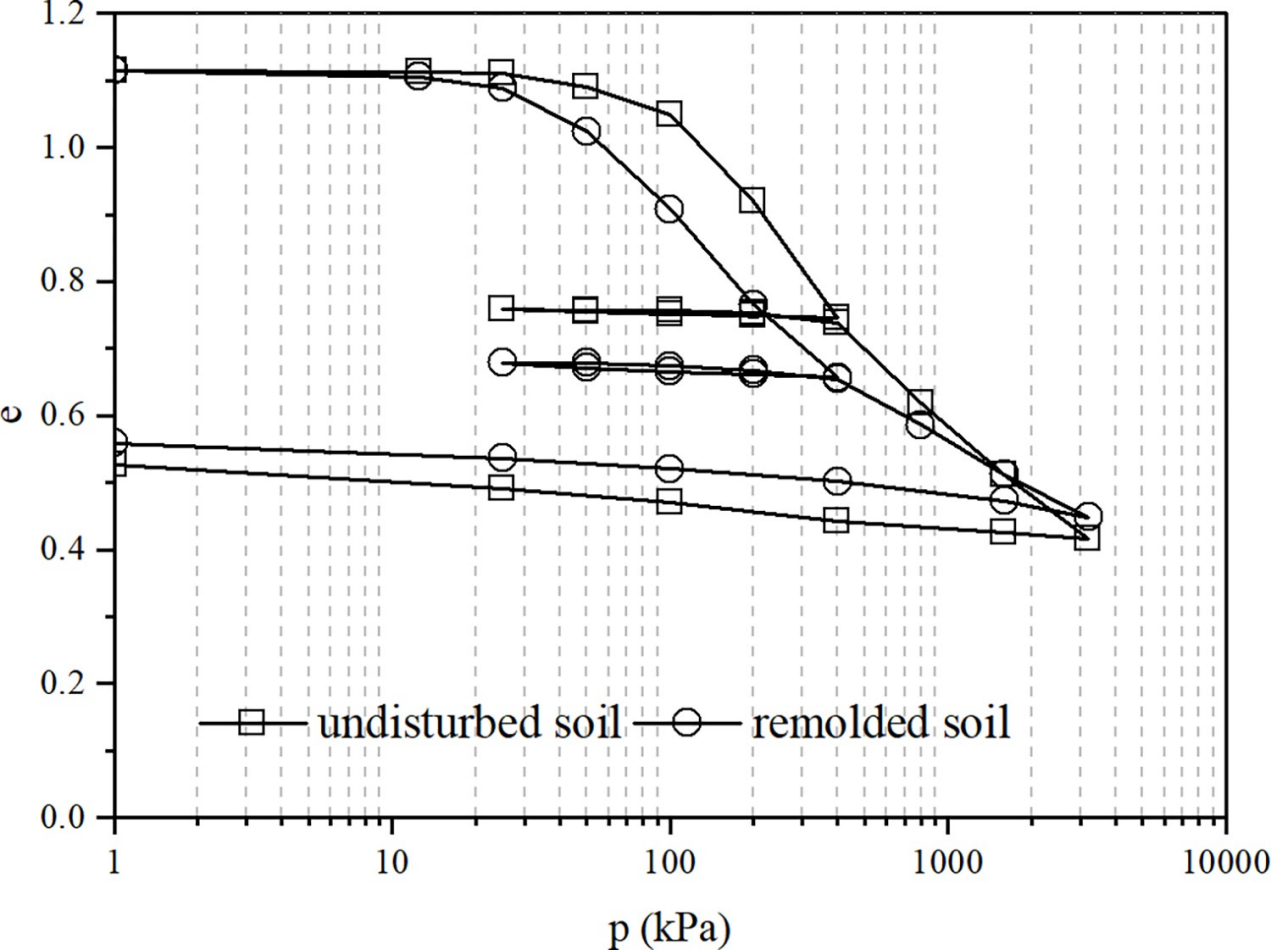
The compression-rebound-recompression test curves of undisturbed and remolded soil.

Fig 3 shows the compression-rebound test curves of undisturbed and remolded soil samples, and Fig 4 shows the compression-rebound-recompression test curves of undisturbed and remolded soil samples, respectively. Fig 3 shows that the compression curve of the undisturbed sample is not significantly different from that of the remolded sample in the initial compression stage with low pressure (*p*≤25*kPa*). At this time, the structural properties of the undisturbed sample are not fully developed, so the difference between the two samples is not significant. As the pressure continues to increase, the structural properties of the undisturbed soil gradually take effect. At 100-400kPa pressure, the structural properties are fully developed, and the compression curve of the undisturbed sample is significantly higher than that of the remolded sample. The difference in pore ratio between the two is 0.131 and remains unchanged. When the pressure exceeds 400kPa, the soil structure gradually damages and weakens, and the compression curves of the undisturbed and remolded samples gradually approaches each other. When the pressure reaches 998kPa, the soil structure is lost, and the compression curves of the undisturbed and remolded samples intersect.

The patterns of the curves in Fig 4 are similar to those in Fig 3. It can be seen that the structure of the undisturbed sample has recovered after the compression-rebound. Therefore, when the pressure of the undisturbed sample is greater than 400kPa, the position of the recompression curve of the undisturbed sample is significantly higher than that of the continuous compression curve; also, the recompression curve of the undisturbed and remolded sample extends beyond until the pressure is 1578kPa before intersecting.

### 3.2 Scanning electron microscope test

From the image presentation elements of the scanning electron microscope results, the skeleton structure of the image magnified by 2000 times is much clear and more comprehensive. Therefore, the image magnified by 2000 times is used for analysis.

**Fig 5:**
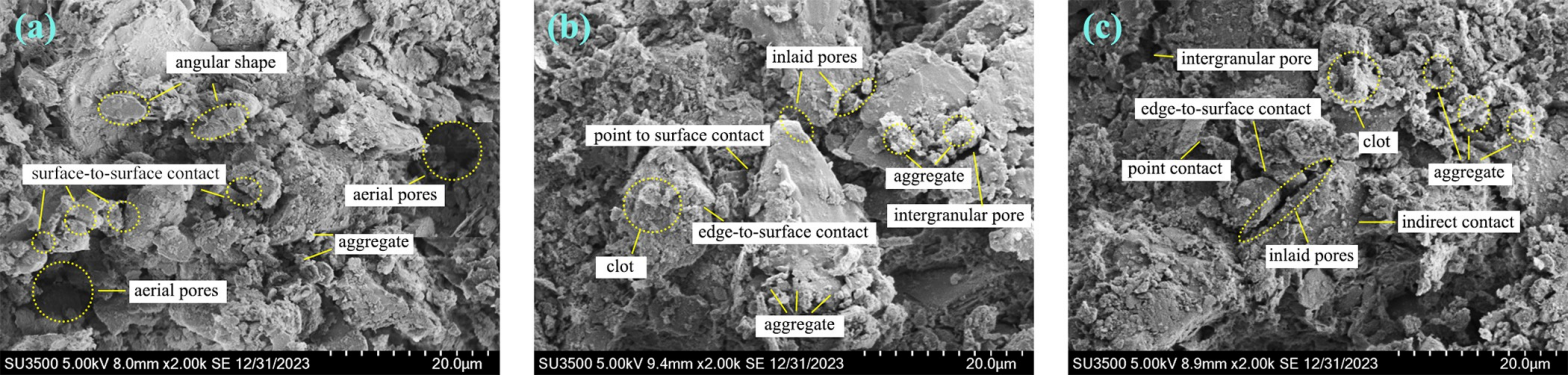
Scanning electron microscopy images of undisturbed soil in various states (magnified by 2000 times). **(**a) Initial state. (b) Compression-rebound state. (c) Compression-rebound-compression state.

Fig 5 shows that the microstructure of soil samples has undergone certain changes under different states. Firstly, the undisturbed loess is mainly angular or rounded in shape, with a certain amount of aggregate present in it. After compression and rebound, the clay minerals between the particles of the soil sample are reduced, some particles aggregate into larger clumps, while others separate and break down, forming small particles. After rebound and compression, the soil sample has more small particles, which are embedded and arranged between pores, enhancing the connection strength of the soil. Secondly, compared to the undisturbed loess, there are relatively less pores formed due to lack of particles in the compression-rebound samples and compression-rebound-recompression samples, while there are relatively more particle embedded pores and intergranular pores, which ensures that the soil sample structure will be more stable. Finally, the contact mode between soil particles has also undergone certain changes. As compression progresses, the contact form of some soil particles gradually changes from surface-surface contact to edge surface contact, and finally develops into edge-edge or point-surface contact. The main reason is that during the compression process of the soil sample, as the load continues to increase, the soil skeleton particles move, and a large number of aggregate particles are decomposed into debris particles and filled in the large pores, causing changes in the contact mode between some particles.

During the rebound process, due to the thixotropy of the soil, the connection force generated by the double electric layer around the loess particles and the suction force at the water air interface will be partially restored. In addition, the increase in embedded pores after compression leads to a larger contact area between particles and a higher degree of cementation, resulting in a stable and strong soil sample structure. After rebound and compression, some soil particles change their morphology from angular to aggregate and agglomerate, and the number and area of pores decrease. Overall, the contact mode of soil particles is mostly dispersed and embedded arrangement. From Fig 5C, it can be seen that most of the soil samples contain a large amount of cementitious material between particles, with fuzzy and difficult to identify particle contours. These contact arrangements make the loess more stable and denser, resulting in better compressive performance of such soil samples compared to compressed rebound soil samples and undisturbed soil samples.

### 3.3 Direct shear test

#### 3.3.1 Comparative analysis of direct shear test of various soil samples under different vertical pressures

**Fig 6:**
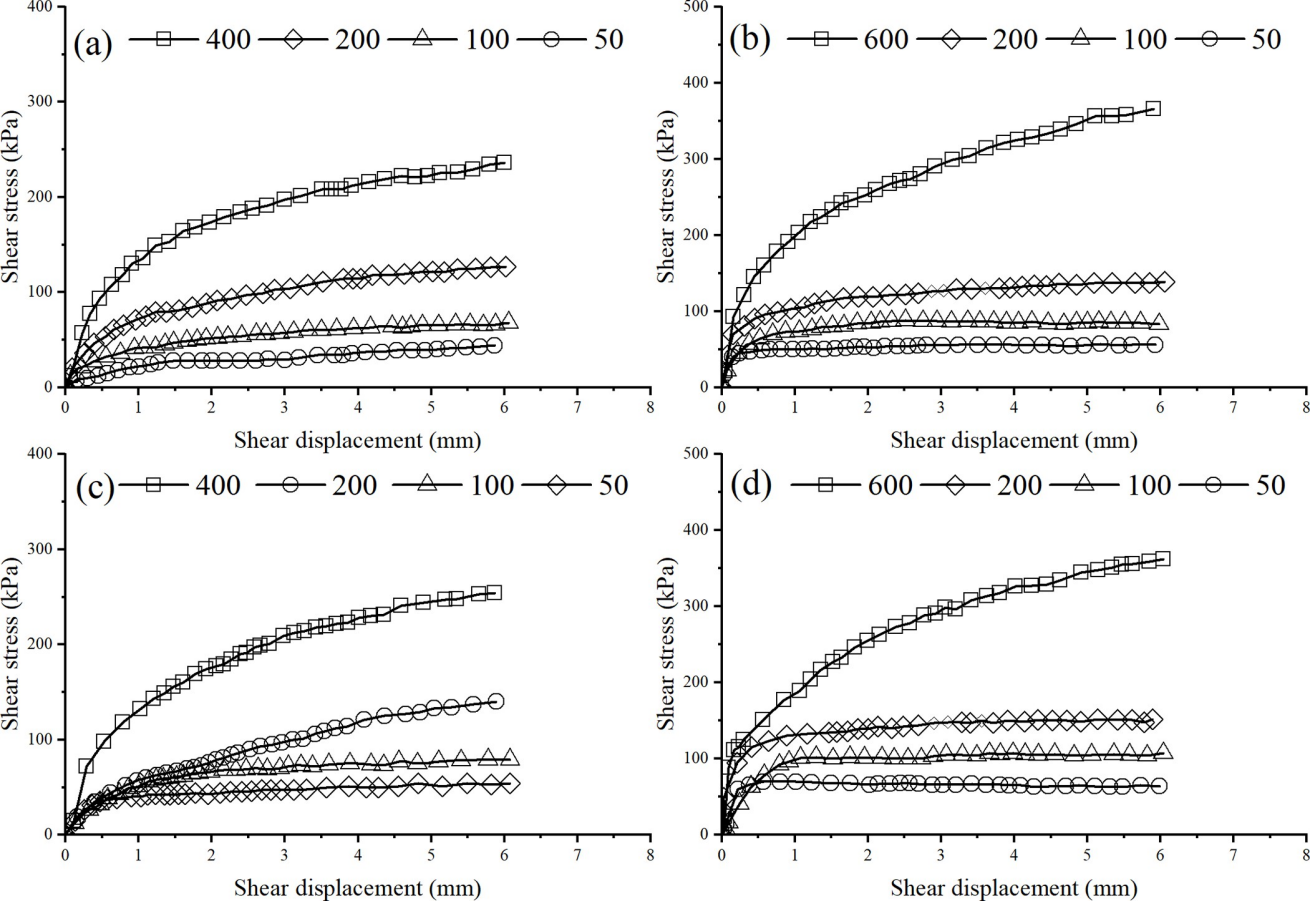
*τ*−*s* (Relationship curves of direct shear test of various soil samples under different vertical pressures. a) cz sample. (b) cc sample. (c) yz sample. (d) yc sample.

Fig 6 presents the *τ*−*s* relationship curves of direct shear tests of various soil samples under different vertical pressures.

As can be seen from Fig 6A and 6C, the peak points of the stress-strain curves for the cz and yz samples at the same moisture content are not obvious at lower vertical pressure values. However, the inflection point of the curve for the yz sample is a little clearer, which may be because some undisturbed soils have a certain degree of natural overconsolidation in stress history. Also, undisturbed soils exhibit similar overconsolidation characteristics due to secondary consolidation, chemical cementation, and drying stress, called quasi-overconsolidation. Due to these reasons, the undisturbed soil exhibits a certain degree of overconsolidation. Therefore, the strength of the yz sample is higher than that of the cz sample under the same vertical pressure. For example, at vertical pressure of 400kPa, the shear strength of yz is 13kPa higher than that of the cz sample. The strengthening phenomenon of the cz curve is more obvious. As shown in Fig 6B and 6D, the inflection points of the relationship curves between the cc and yc samples are more pronounced at low vertical pressure values, and the curves shows a downward trend after the inflection points. After compaction and rebound, the overconsolidation characteristics of loess are more significant. Under the same vertical pressure, the strength of the yc sample is higher than that of the cc sample. For example, at vertical pressure of 200kPa, the shear strength of yz is 19kPa higher than that of cz sample. The work hardening phenomenon of the cc curve is not obvious.

Experimental results have shown that the structure and overconsolidation of soil can improve its strength. However, the structural characteristics of undisturbed loess are significant, and the deformation is relatively small under overconsolidation load compression. Therefore, compared to remolded loess, the compaction degree of overconsolidated original loess will be reduced, and when the soil is compressed, its structure will also be damaged. This indicates that the structure and overconsolidation of soil will weaken each other. However, overconsolidation has a compaction effect, and the structure of the soil can also recover to a certain extent during the rebound process. Therefore, the superposition of the structure and overconsolidation will improve the total strength of the soil. This is the reason why undisturbed overconsolidated soil has the highest strength.

#### 3.3.2 Comparative analysis of direct shear test of various soil samples under the same vertical pressure

**Fig 7:**
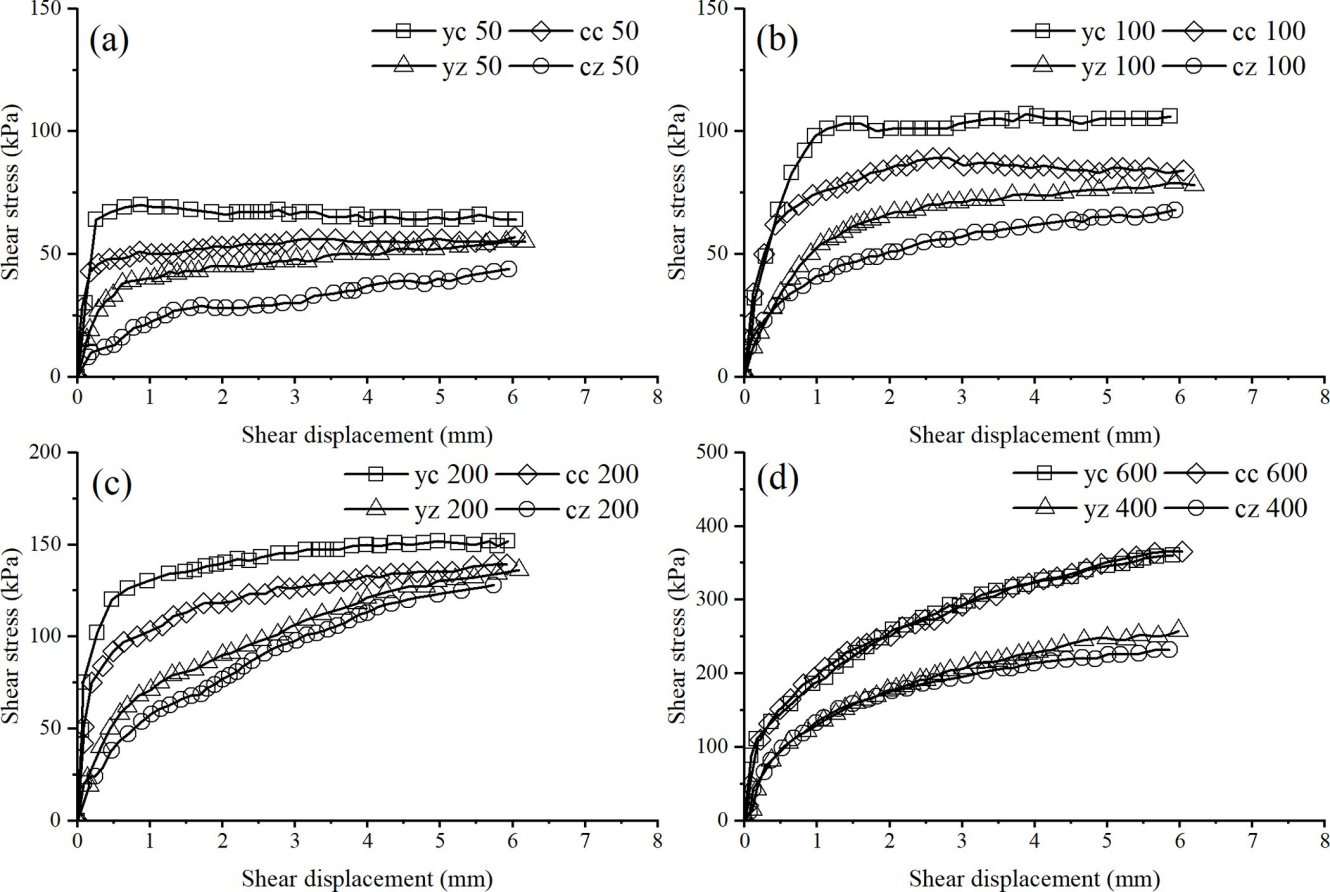
*τ*−*s* relation curves of direct shear test of various soil samples under the same vertical pressure. **(**a) 50kPa. (b) 100kPa. (c) 200kPa. (d) 400kPa and 600kPa.

Fig 7 presents the *τ*−*s* relationship curves of various soil samples under the same vertical pressure in the direct shear test.

As shown in Fig 7, the shear stress of all the soil samples increases with vertical pressure, but the rate of increase in shear stress first increases and then gradually decreases. The magnitude of the shear stress of the four types of soil samples is yc>cc>yz >cz. The strength disparity between the yz and cz samples and between the yc and cc samples is mainly caused by the structure. Also, the application of overconsolidation loads can compress the soil, which can damage the soil structure. The yc sample is weaker than the structure of the yz sample, and the influence of structure on soil strength is accordingly reduced. For the shear strength under a vertical pressure of 50kPa, the yz sample is 35% higher than the cz sample, while the yc sample is only 26% higher than the cc sample.

Since the influence of structural properties is not considered in remolded soils, the strength difference between the cc and cz samples is only related to overconsolidation. However, the strength disparity between the yc and yz samples is influenced by structural properties and overconsolidation. As the structural properties reduce the deformation of the soil caused by overconsolidation, the undisturbed soil is less compact than the remolded soil. Therefore, the effect of overconsolidation on the strength of the undisturbed soil is weaker than that of the remolded soil. At a vertical pressure of 50kPa, the strength of the cc sample increased by 51.3% compared to the cz sample, and the strength of the yc sample increased by only 40% compared to the yz sample.

When the vertical pressure is low, the degree of overconsolidation of the soil is relatively high, and overconsolidation significantly impacts the soil strength. When the vertical pressure is 50kPa, the structure increases the strength of the yz sample by 35% compared to the cz sample and overconsolidation increases the strength of the cc sample by 51.3% compared to the cz sample. As the vertical load increases, the overconsolidation degree of the soil sample decreases, and the effect of overconsolidation on soil strength weakens. At 100kPa vertical pressure, overconsolidation increased the strength of the cc sample by 37.9% compared to the cz sample. In contrast, at 200kPa, overconsolidation only increased the strength of the cc sample by 17.7% compared to the cz sample. When the vertical pressure increased and the overconsolidation degree decreased, the structural influence of the soil became larger. At 50kPa, the strength of the yc sample increased by 40% more than the yz sample, while that of the yc sample increased by 44.6% over that of the yz sample at 100kPa. However, when the vertical pressure was 100kPa, the structural strength of the yz sample increased by 21% compared with the cz sample, while the structural strength of the yz sample increased by only 7% compared with the cz sample at 200kPa. Therefore, with the continuous increase in vertical pressure, the structural damage is more serious, and the overconsolidation degree is also weakened. The effects of both factors on the soil strength are reduced, and the difference of influence is gradually approaching. It is also clear from Fig 7D that as the vertical pressure continues to increase, the influence of structure and overconsolidation on the soil strength becomes less and less. When the consolidation pressure increases to 600kPa, the structure is fully destroyed and the overconsolidation degree is reduced to 1, and both factors have lost their effect on the soil strength.

#### 3.3.3 Discussion of the coupling influence law of structure and overconsolidation

**Fig 8:**
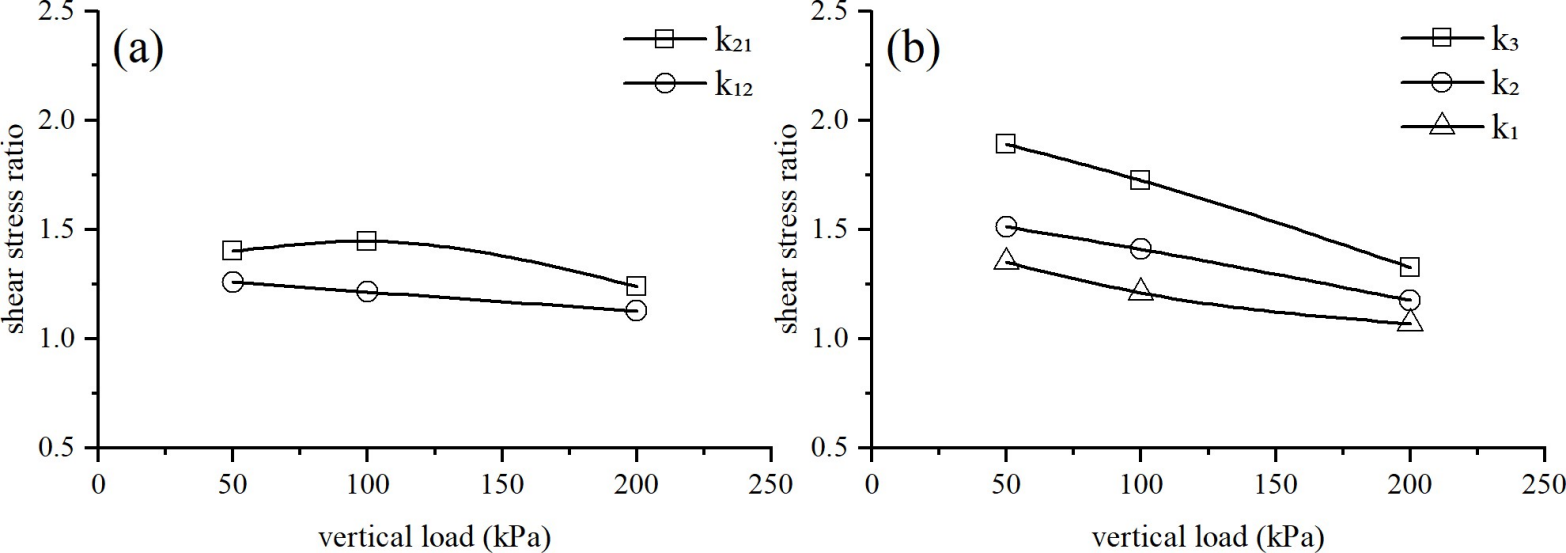
Shear stress ratio curves.

In order to further explore the coupling effect of overconsolidation and soil structure on the stress-strain relationship of loess, the shear stress ratio for various samples under the same vertical pressure was determined. The shear stress ratio curve is shown in Fig 8. For the convenience of analysis, yc/cz is used in the text to represent the shear stress ratio between the overconsolidated undisturbed soil sample and the normal remolded soil sample. Likewise, yz/cz, yc/cc, cc/cz, and yc/yz represent the shear stress ratio between different soil samples. The value of yz/cz is *k*_*1*_, which can reflect the influence of soil structure on strength. The value of cc/cz is *k*_*2*_, which can reflect the influence of overconsolidation on soil strength. The value of yc/cc is *k*_*12*_, which can reflect the influence of structure on overconsolidated soil. The value of yc/yz is *k*_*21*_, which can reflect the influence of overconsolidation on structural soil. The value of yc/cz is *k*_*3*_, which can reflect the influence of structural and overconsolidation coupling on soil strength.

As shown in Fig 8, the *k*_12_ curve initially slightly rises and then slowly decreases, indicating that the influence of structure on overconsolidation first increases and then decreases. When the vertical load is greater than the pre-consolidation pressure, the structure of the undisturbed soil is significantly damaged and weakened, and the influence of structural properties on overconsolidation is correspondingly reduced.

The *k*_21_ curve shows a linear and slowly decreasing trend. As the vertical pressure increases, the degree of overconsolidation continues to decrease, and the effect of overconsolidation on structural properties constantly decreases. The combined effect of over consolidation and structural characteristics can maximize the strength of soil. However, due to the mutual interference and weakening of these two factors, the strength increase value caused by the combined effect is smaller than the simple sum of the increase values caused by the individual effects of the two factors.

#### 3.3.4 The relationship between the shear stress ratio and the vertical load

Asaoka et al. divided the state of soil into four possibilities, i.e., normal consolidated structural soil, over-consolidated structural soil, over-consolidated remolded soil, and normal consolidated remolded soil [43]. It was shown that with plastic deformation, the soil in the first three states eventually becomes the final normal consolidated remolded soil. Therefore, there is an intrinsic relationship between normal consolidated remolded soil and the other three soil states, which can be used as a reference for calculating the other three soil states.

In order to study the influence of structure and overconsolidation on the shear strength of loess, empirical formulas for the shear stress ratio *k* and vertical load *p* of different types of specimens were established based on experimental results, and conversion formula for various *k* was also provided, as shown in Table 4. The proposed formula can reflect the relationship between structure, overconsolidation, and their coupling.

**Table 4:** Analysis of fitting results.

Type	Fitting formula	Parameter values	Correlation coefficient/R^2^
*k*_*1*_ = yz/cz	*k*_1,2,3_ = *mp*+*n*	m = -0.0038 n = 2.09	0.998
*k*_*2*_ = cc/cz		m = -0.0023 n = 1.63	0.999
*k*_*3*_ = yc/cz		m = -0.0018 n = 1.42	0.964
The relationship between *k₁ k₂* and *k₃*	$k_{3}=\frac{k_{1}k_{2}}{\sqrt{e}}$	None	0.956

Where *m* and *n* are fitting coefficients, and *e* is the corresponding void ratio under different vertical load of undisturbed loess. The shear stress ratios under different vertical loads were selected for the fitting analysis. All three fitting formulas can reflect the relationship between shear stress ratio and vertical pressure to a certain extent and have a high correlation.

**Fig 9:**
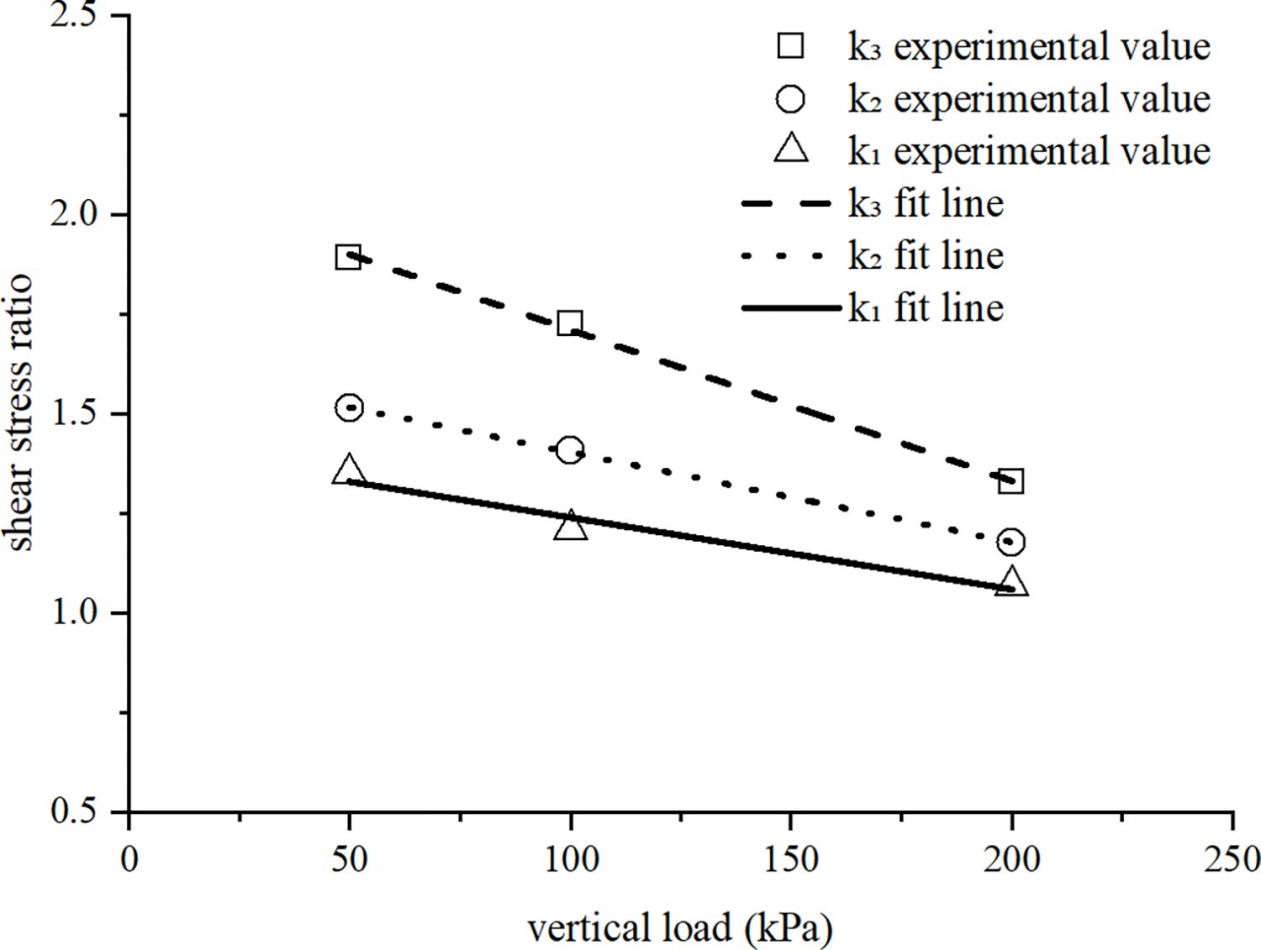
The calculated and experimental values of *k*_*1*_, *k*_*2*_ and *k*_*3*_.

Fig 9 compares the calculated and experimental values of *k₁*, *k₂*, and *k*_*3*_. The difference between the two types of values is small and the trend is consistent. The variation trends of *k₁*, *k₂*, and *k*_*3*_ curves are similar, and the three shear stress ratios all decrease with the increase of vertical pressure. Therefore, under low vertical pressure, the structure and overconsolidation have a significant effect on the strength of the soil. Under higher vertical pressure, the effect of structure and overconsolidation on strength decreases and eventually disappears. The *k*_*3*_ curve is located above the *k₁* and *k₂* curves, indicating that both structure and overconsolidation increase soil strength, and the coupling effect of the two factors further increases the shear strength of the soil. The *k*_*2*_ curve is located above the *k*_*1*_ curve, indicating that overconsolidation can increase soil strength more effectively than structure. As the vertical load increases, the structural damage of the soil increases, and the degree of overconsolidation also decreases, thus, the difference between the three curves gradually disappears.

**Fig 10:**
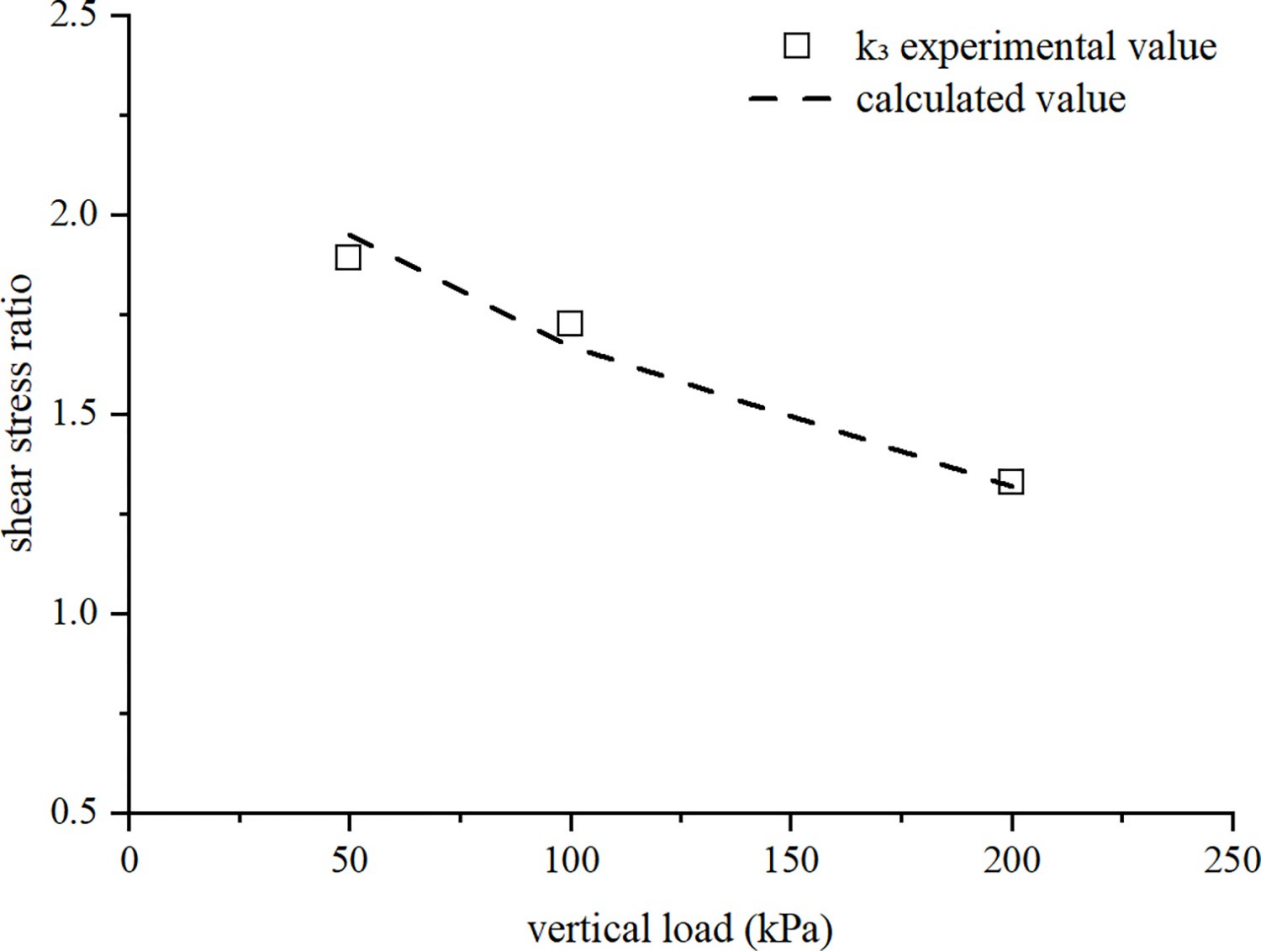
Comparison of *k*_*3*_ values calculated from *k*_*1*_ and *k*_*2*_ with experimental values.

Fig 10 shows a comparison between the calculated and experimental values of *k₃*, and the *k*_*3*_ is calculated from *k*_*1*_ and *k*_*2*_. The calculated values are relatively close to the experimental values, and the trend is consistent. This indicates the rationality of the formula proposed in this article. The results indicate that both structural and overconsolidation positively affect soil strength, with overconsolidation having a relatively greater effect. The respective effects are diminished when the two factors are added to each other.

#### 3.3.5 Summary of direct shear test in *τ*−*σ* plane

**Fig 11:**
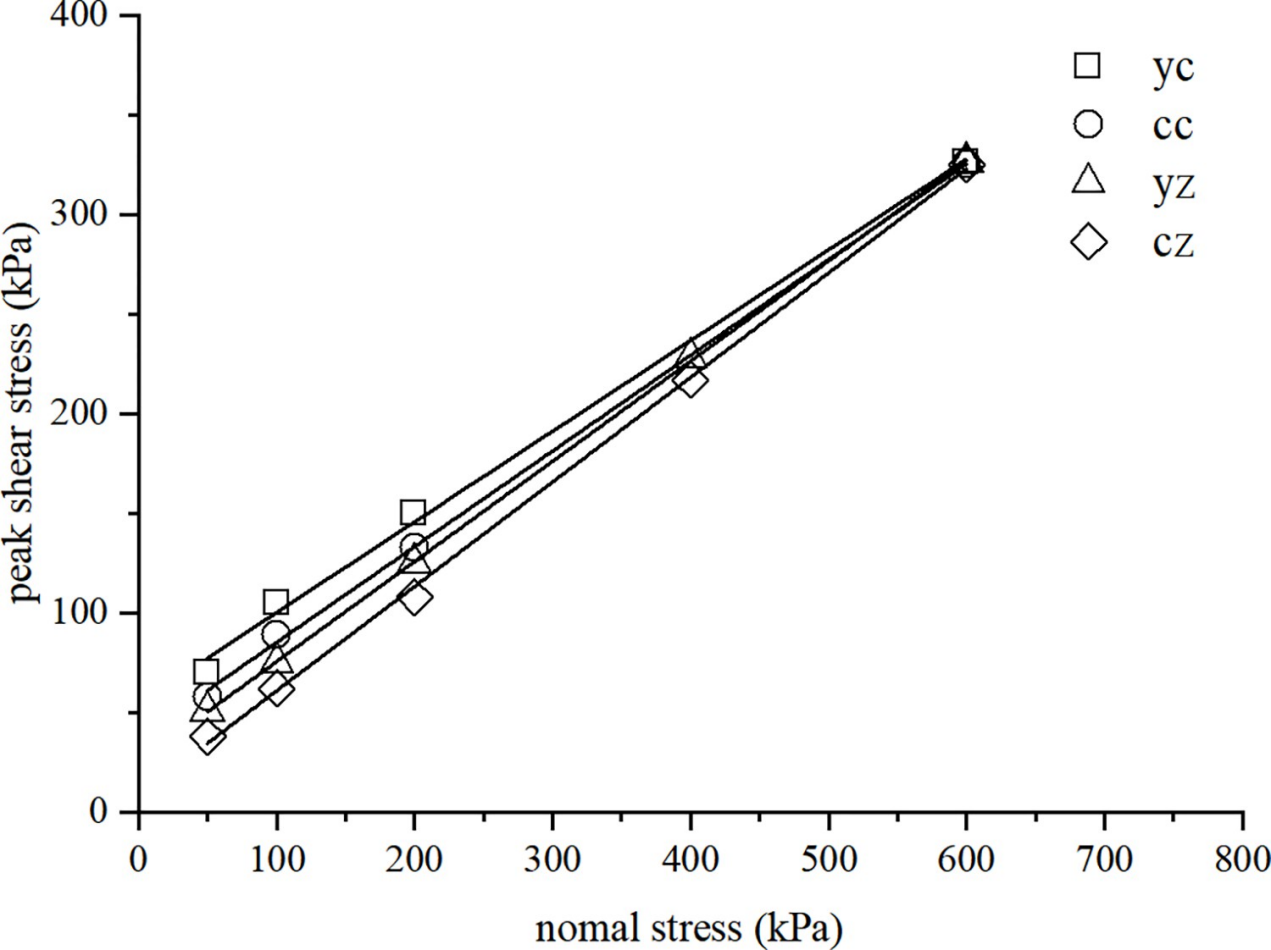
Curves of various soil samples in direct shear tests.

Fig 11 shows the *τ*−*σ* curves of various types of soil sample in direct shear tests. The results of various tests can be approximated as a linear relationship. The *c* and *φ* values of different soil samples are presented in Table 5.

**Table 5:** Summary table of *c* and *φ* values of test soil samples.

Description	cz	cc	yz	yc
*C/*kPa	8.5	37.23	25.35	54.73
*φ/°*	27.65	25.64	26.66	24.46

The difference in *c* values of different soil samples is much greater than that of the *φ* value. The *φ* value of the yz sample is relatively close to that of the cz sample, while the *c* value of the yz sample is 2.98 times that of the cz sample; The *φ* value of the yc sample and cc sample is also relatively close, but the *c* value of the yc sample is only 1.47 times that of cc sample. Hence, the influence of structure on the *c* value of soil is much greater than the *φ* value.

## 4 Conclusions

In this paper, the effects of soil structure and overconsolidation on the mechanical properties of soil under natural moisture content have been studied by compression test, compression-rebound test, scanning electron microscopy observation, and direct shear tests. Undisturbed loess, remolded loess, overconsolidated undisturbed loess, and overconsolidated remolded loess were investigated in this work. The following conclusions are obtained from the study.

1. The compression deformation of the undisturbed loess is smaller than that of the remolded loess, and the corresponding compression curve is higher than that of the remolded loess. The turning point of the curve is more pronounced. Although the compression process of overconsolidation can cause damage to the structure, during the rebound process of overconsolidation, the structure can be partially restored.
2. Under the same strain conditions, structural effects determine that the shear strength of undisturbed loess is greater than that of remolded loess. The overconsolidation effect determines that the strength of over consolidated loess is greater than that of normally consolidated loess. The magnitude of the shear stress of the four types of soil samples varies in the order yc>cc>yz >cz. The structure and overconsolidation can enhance the overall strength of the soil, but the effects of these two factors also interfere and weaken each other. The increase in soil strength caused by the coupling effect is smaller than the sum of the strength increase caused by the two factors. The microscopic observation of electron microscopy is consistent with the macroscopic experimental rules.
3. According to the experimental results and analysis, the empirical formula of the influence of overconsolidation, structure, and the coupling effect of the two on soil strength is presented. The calculated results of the formula are highly consistent with the experimental data, and have good rationality and accuracy.

The effect of soil structure and overconsolidation on its mechanical properties is a complex and interesting issue. The conclusion of this paper is obtained by studying loess under certain conditions. In the future, the research can be conducted on the coupling effects of structure and overconsolidation under other conditions, such as variable water content and dry density, in order to gain a more comprehensive and in-depth understanding of the essence of this issue.

## Supporting information

S1 Dataset (PDF)
