# Supplementary material for: Study on coupling effect of soil structure and overconsolidation on mechanical properties of loess
Source: PLoS One. 2024 Mar 13;19(3):e0298653. doi: 10.1371/journal.pone.0298653 (PMC10936806; doi:10.1371/journal.pone.0298653)
Supplement: S1 Dataset — (PDF) [file pone.0298653.s001.pdf]

Compression rebound

	cz sample	yz sample
p	e	e
1	1.115	1.115
12.5	1.09	1.1118275
25	1.06	1.1097125
50	1.000285	1.087505
100	0.897155	1.036745
200	0.7665075	0.909845
400	0.6497	0.753335
800	0.56818	0.5651
1600	0.4994425	0.42128
3200	0.422245	0.296495
1600	0.439165	0.3208175
400	0.4656025	0.3504275
100	0.4846375	0.3694625
25	0.5005	0.3800375
1	0.5340875	0.419165

compression-rebound-compression

	cz sample	yz sample
p	e	e
1	1.115	1.115
12.5	1.1033675	1.112885
25	1.074815	1.11077
50	1.01348	1.08962
100	0.90773	1.049435
200	0.766025	0.91989125
400	0.6571025	0.7459325
200	0.6613325	0.749105
100	0.6655625	0.75174875
50	0.67085	0.7543925
25	0.6782525	0.7586225
50	0.6782525	0.757565
100	0.6740225	0.7565075
200	0.6676775	0.7522775
400	0.65393	0.73800125
800	0.58625	0.61850375
1600	0.512225	0.5111675
3200	0.448775	0.4159925
1600	0.4730975	0.42551
400	0.50165	0.44190125
100	0.520685	0.469925
25	0.5365475	0.49054625
1	0.558755	0.5259725

50kPa

yc sample		cc sample		yz sample	
Shear displacement	Shear stress	Shear displacement	Shear stress	Shear displacement	Shear stress
0.11	30	0.06	28	0.17	19
0.26	64	0.18	43	0.29	27
0.45	67	0.29	46	0.39	31
0.65	69	0.44	48	0.49	33
0.87	70	0.59	48	0.62	38
1.08	69	0.72	49	0.75	39
1.3	69	0.86	51	0.95	40
1.57	68	1.02	50	1.06	40
1.86	67	1.16	50	1.16	41
2.05	66	1.29	50	1.27	43
2.2	67	1.46	51	1.37	42
2.34	67	1.6	52	1.47	43
2.49	67	1.73	52	1.57	43
2.6	67	1.86	53	1.67	43
2.77	68	1.99	53	1.83	45
2.92	66	2.14	53	2	45
3.07	67	2.29	54	2.16	45
3.29	67	2.45	54	2.3	45
3.48	65	2.59	54	2.46	46
3.68	65	2.72	54	2.58	46
3.86	66	2.88	55	2.74	47
3.98	64	3.08	56	2.86	47
4.14	65	3.32	56	3.02	48
4.35	65	3.56	56	3.17	47
4.57	64	3.8	55	3.39	49
4.74	64	4.04	55	3.67	50
4.94	65	4.25	55	3.96	50
5.17	64	4.44	55	4.17	50
5.38	65	4.63	55	4.37	52
5.55	66	4.82	56	4.63	52
5.84	64	5.02	56	4.93	52
6.05	64	5.27	55	5.26	53
		5.49	55	5.63	54
		5.68	55	5.91	55
		6.03	57	6.17	55

50kPa

cz sample

Shear displacement	Shear stress
0.19	10
0.38	12
0.51	13
0.62	16
0.78	20
0.92	21
1.05	23
1.22	25
1.33	27
1.56	28
1.71	29
1.9	28
2.05	28
2.24	28
2.47	29
2.65	29
2.83	30
3.07	30
3.26	33
3.54	34
3.72	35
3.82	35
3.98	37
4.16	38
4.41	39
4.61	39
4.79	38
4.97	40
5.13	39
5.34	41
5.54	42
5.75	43
5.95	44

100kPa

yc sample		cc sample		yz sample	
Shear displacement	Shear stress	Shear displacement	Shear stress	Shear displacement	Shear stress
0.14	32	0.12	34	0.21	18
0.29	49	0.27	50	0.39	28
0.46	68	0.42	62	0.5	34
0.65	83	0.58	67	0.64	40
0.84	92	0.74	70	0.75	45
0.97	98	0.94	74	0.91	50
1.14	101	1.12	76	1.05	54
1.37	103	1.28	77	1.17	56
1.61	103	1.41	79	1.27	57
1.82	100	1.56	80	1.36	59
2.03	101	1.73	83	1.47	61
2.26	101	1.91	84	1.55	62
2.43	101	2.09	86	1.65	63
2.6	101	2.23	86	1.75	64
2.78	101	2.38	88	1.87	65
2.95	103	2.6	89	2.06	67
3.14	104	2.82	89	2.23	67
3.35	105	3.02	86	2.37	68
3.55	105	3.21	87	2.53	70
3.7	104	3.43	87	2.66	70
3.88	107	3.59	86	2.81	71
4.04	106	3.77	86	2.96	71
4.22	105	3.94	85	3.12	72
4.4	105	4.12	86	3.27	72
4.64	103	4.32	85	3.45	72
4.89	105	4.54	84	3.69	74
5.15	105	4.73	84	3.99	74
5.44	105	4.89	83	4.26	74
5.66	105	5.06	85	4.41	75
5.87	106	5.21	85	4.71	76
		5.38	84	4.89	76
		5.57	85	5.21	77
		5.82	83	5.51	77
		6.05	84	5.92	79
				6.2	78

100kPa
cz sample

Shear displacement	Shear stress
0.11	16
0.27	23
0.44	29
0.63	34
0.81	37
0.99	41
1.14	42
1.28	45
1.39	46
1.55	47
1.72	49
1.82	49
2	51
2.09	51
2.28	53
2.47	55
2.68	56
2.8	56
2.99	57
3.18	59
3.38	59
3.55	60
3.79	61
4.02	62
4.28	63
4.53	64
4.65	63
4.78	65
4.95	65
5.16	66
5.39	65
5.61	66
5.79	67
5.94	68

200kPa					
yc sample		cc sample		yz sample	
Shear displacement	Shear stress	Shear displacement	Shear stress	Shear displacement	Shear stress
0.08	75	0.09	51	0.14	23
0.27	102	0.2	75	0.32	40
0.48	120	0.35	84	0.45	50
0.7	126	0.49	92	0.59	58
0.97	130	0.64	97	0.69	62
1.26	134	0.82	100	0.85	68
1.49	135	1	103	1.01	71
1.62	136	1.17	106	1.14	75
1.77	138	1.32	111	1.31	79
1.92	139	1.5	113	1.41	80
2.04	140	1.74	118	1.61	82
2.21	142	1.95	118	1.8	86
2.37	141	2.13	119	2	90
2.53	143	2.36	123	2.12	91
2.77	145	2.58	123	2.33	95
2.96	145	2.76	127	2.54	98
3.15	147	2.92	126	2.76	101
3.33	147	3.1	127	2.92	105
3.44	147	3.26	128	3.15	109
3.59	147	3.47	129	3.37	112
3.79	149	3.67	130	3.63	115
3.99	150	3.84	131	3.86	118
4.17	149	3.99	133	3.99	121
4.38	151	4.16	132	4.25	124
4.58	150	4.32	134	4.56	127
4.78	151	4.49	134	4.8	127
4.97	152	4.68	135	4.96	130
5.25	151	4.96	135	5.21	131
5.47	150	5.17	135	5.47	132
5.68	152	5.45	138	5.79	134
5.79	149	5.73	139	6.09	136
5.94	152	5.92	139		

200kPa
cz sample

Shear displacement	Shear stress
0.24	24
0.47	38
0.7	47
0.89	54
1.02	58
1.15	61
1.28	63
1.43	66
1.57	68
1.71	69
1.79	72
1.89	74
2	77
2.1	79
2.2	81
2.29	84
2.41	87
2.58	91
2.73	94
2.87	96
3.02	98
3.16	101
3.3	102
3.45	104
3.59	106
3.75	109
3.89	112
3.99	113
4.14	116
4.33	118
4.56	120
4.97	123
5.3	125
5.74	128

400kPa

yz sample		cz sample	
Shear displacement	Shear stress	Shear displacement	Shear stress
0.17	42	0.12	43
0.37	82	0.27	66
0.61	106	0.37	82
0.86	122	0.52	99
1.09	136	0.68	112
1.26	145	0.81	119
1.39	152	0.98	133
1.54	160	1.09	140
1.66	164	1.3	152
1.82	169	1.49	159
1.97	177	1.7	165
2.09	179	1.82	169
2.19	183	2.03	176
2.3	186	2.24	180
2.42	191	2.46	186
2.5	191	2.62	189
2.62	196	2.84	193
2.7	199	3.06	197
2.83	204	3.32	203
3.02	207	3.57	207
3.18	214	3.68	207
3.33	215	3.82	211
3.49	217	3.99	214
3.61	220	4.22	217
3.78	225	4.44	219
3.91	227	4.66	221
4.05	229	4.84	220
4.23	232	5.01	226
4.4	238	5.18	226
4.68	245	5.44	227
5	248	5.65	232
5.26	246	5.86	232
5.43	252		
5.7	250		
5.98	258		

600kPa			
yc sample		cc sample	
Shear displacement	Shear stress	Shear displacement	Shear stress
0.17	111	0.07	49
0.37	134	0.22	110
0.64	158	0.34	132
0.95	186	0.49	152
1.12	194	0.63	165
1.27	209	0.81	185
1.4	218	0.96	194
1.56	228	1.11	208
1.69	236	1.29	219
1.89	248	1.41	229
2.05	259	1.56	235
2.23	265	1.68	240
2.46	275	1.82	247
2.63	281	2.01	251
2.83	293	2.17	263
2.97	291	2.35	268
3.09	298	2.49	273
3.27	307	2.61	273
3.47	312	2.79	282
3.7	317	3	292
3.87	320	3.24	301
4.1	326	3.47	306
4.3	329	3.7	317
4.51	331	3.92	322
4.71	340	4.12	328
5.01	346	4.31	330
5.22	349	4.51	335
5.41	353	4.71	342
5.51	356	4.92	349
5.69	359	5.2	355
5.91	360	5.4	359
		5.65	364
		6.03	365
